# Potential Effectiveness of Point-of-Use Filtration to Address Risks to Drinking Water in the United States

**DOI:** 10.1177/1178630217746997

**Published:** 2017-12-12

**Authors:** Kathleen Ward Brown, Bemnet Gessesse, Lindsey J Butler, David L MacIntosh

**Affiliations:** 1 Environmental Health & Engineering Inc., Needham, MA, USA; 2 Department of Environmental Health, Boston University School of Public Health, Boston, MA, USA; 3 Department of Environmental Health, Harvard T.H. Chan School of Public Health, Boston, MA, USA

**Keywords:** Point-of-use (POU) filter, drinking water treatment, faucet-mount filter, filtration, NSF, Flint

## Abstract

Numerous contemporary incidents demonstrate that conventional control strategies for municipal tap water have limited ability to mitigate exposures to chemicals whose sources are within distribution systems, such as lead, and chemicals that are not removed by standard treatment technologies, such as perfluorooctanoic acid (PFOA)/perfluorooctanesulfonic acid (PFOS). In these situations, point-of-use (POU) controls may be effective in mitigating exposures and managing health risks of chemicals in drinking water, but their potential utility has not been extensively examined. As an initial effort to fill this information gap, we conducted a critical review and analysis of the existing literature and data on the effectiveness of POU drinking water treatment technologies for reducing chemical contaminants commonly found in tap water in the United States. We found that many types of water treatment devices available to consumers in the United States have undergone laboratory testing and often certification for removal of chemical contaminants in tap water, but in most cases their efficacy in actual use has yet to be well characterized. In addition, the few studies of POU devices while “in use” focus on traditional contaminants regulated under the Safe Drinking Water Act, but do not generally consider nontraditional contaminants of concern, such as certain novel human carcinogens, industrial chemicals, pesticides, pharmaceuticals, personal care products, and flame retardants. Nevertheless, the limited information available at present suggests that POU devices can be highly effective when used prophylactically and when deployed in response to contamination incidents. Based on these findings, we identify future areas of research for assessing the ability of POU filters to reduce health-related chemical contaminants distributed through public water systems and private wells.

## Introduction

The US Safe Drinking Water Act (SDWA), passed in 1974 and amended in 1986 and 1996, authorizes the US Environmental Protection Agency (USEPA) to set health-based standards for contaminants in public drinking water systems. Public drinking water systems are defined as water systems that have 15 service connections or more or serve at least 25 people daily for 60 days or more out of the year. Under the authority of the SDWA, USEPA establishes National Primary Drinking Water Regulations, which are health-based enforceable upper limits for concentrations of selected contaminants measured in drinking water, ie, standards. USEPA currently has primary standards for more than 75 contaminants and an extensive Contaminant Candidate List (CCL), substances that are under review for potential future regulation. Using the Unregulated Contaminant Monitoring Rule (UCMR), USEPA can require public water systems (PSWs) to monitor certain contaminants not otherwise regulated to assess their occurrence and levels in drinking water.^1^

Even with these standards and other regulations in place, several contemporary incidents have highlighted the vulnerability of tap water in the United States to chemical contamination. For example, lead continues to be a prevalent concern despite the long-held and extensive knowledge about its sources in public water supplies and toxicity.^2^ More recently, perfluorooctanoic acid (PFOA) has been detected in tap water of numerous public drinking water systems at levels previously found to be associated with kidney and testicular cancer in similarly exposed populations.^3^

The principal safeguards for tap water delivered by PSWs are located at centralized treatment facilities, which have limited ability to control contamination that arises in downstream distribution systems, such as lead, and to remove organic compounds such as PFOA.^2,4–6^ The aging infrastructure in the United States contributes to the potential for contaminants to enter tap water downstream of drinking water treatment facilities. According to USEPA, 30% of pipes in water systems serving more than 100 000 people are between 40 and 80 years old and approximately 9% of pipes in those systems are even older.^7^ The quality of drinking water produced by private wells is subject to many of the same threats as municipal water supplies.

Given the limitations of current centralized treatment systems to protect drinking water quality, filtration of water at homes, places of work, schools, and other points of use (POUs) may be beneficial. However, the potential for POU filtration technologies to contribute to drinking water safety has yet to be characterized in the context of public health. The objective of this article is to begin to fill that knowledge gap. To that end, we (1) present a critical review of the existing data and scientific literature on the effectiveness of POU filtration devices, including their use to control lead in Flint, Michigan, and (2) examine data from the USEPA’s Enforcement and Compliance History Online (ECHO) database to assess the scope of SDWA violations in the United States and the potential opportunity for POU technologies to reduce consumer exposures in those settings.

## Methods

### Literature search

We conducted a search of the scientific literature to identify studies of the efficacy of POU filters in actual use. We searched Web of Science, PubMed, JSTOR, and Google Scholar databases for relevant published articles using the following search terms or variants of the term: “point of use” and “drinking water treatment” OR filtration OR purification OR device OR residential OR tap OR system OR homes OR POU. Most studies of residence-level drinking water filtration/treatment have been conducted in developing countries and have focused on microbiological contaminants. As the focus of this review is on the evaluation of POU filters available to US consumers, we used the ‘NOT’ function in JSTOR and Google Scholar databases to further narrow results and excluded the following search terms (Africa, developing, ceramic, Nepal, Asia, epidemiology, and hospital). We limited our results to articles published in the past 2 decades, 1996-2016. We screened the titles and/or abstracts of articles for eligibility and reviewed full texts of the relevant abstracts. References provided in eligible articles were screened to identify additional studies of interest. Studies from peer-reviewed journals were included in our review if they met all of the following: (1) POU performance evaluation study, (2) conducted in United States or Canada, (3) published in English, (4) investigated chemical contaminant removal by POU drinking water treatment devices/technologies for residential use, (5) conducted lab-scale and/or field evaluation studies of POU devices, and (6) provided performance data for contaminants tested.

### Data analyses

While conducting the literature search, we identified a large set of relevant data published by USEPA that can be used to evaluate the efficacy of POU filtration for removal of lead and other elements from tap water.^8^ We downloaded the data from the on-line USEPA database and carried out several evaluations of the filter effectiveness.

POU faucet-mount filters approved for lead removal and other contaminants by an independent review body, NSF International (nsf.org), were distributed to residents in the Flint, MI, service area to treat residual Pb contamination after the city switched the water supply and introduced system treatments and upgrades. The data published by USEPA included drinking water samples collected in 238 residences, 1 church, and 34 commercial properties in Flint.^8^ Where possible, 3 samples were collected at each home or facility: (1) with the POU filter in-line using the existing cartridge in place at the time of sampling (“used filter”), (2) with the POU filtration device removed (“no filter”), and (3) with a new replacement filter cartridge inserted into the POU filtration device (“new filter”). These samples were collected sequentially at one or more sinks in each home or facility. Samples were analyzed for Al, Cd, Ca, Cr, Cu, Fe, Pb, Mg, Mn, Ni, K, Na, and Zn using USEPA Method 200.8. Six samples were analyzed for tin but were not included in this analysis due to the very limited sample size.

For our analysis, we matched the used filter and no filter data by property and house identification codes, which yielded 208 observations for 13 elements. We then generated summary statistics of element concentrations in filtered and nonfiltered water and the differences between the two. Next, we used Wilcoxon signed-rank 2-sample paired tests to determine whether differences between filtered and nonfiltered water were statistically different from 0 at .05 level of significance. We also assessed the differences between used and new replacement cartridges in the POU filters for the 161 observations that had both measures to determine whether concentrations of elements vary significantly between used and new filters. Data processing, summary statistics, and Wilcoxon tests were done using R.^9^

### Assessment of USEPA violation data

We queried the USEPA ECHO database to assess the scope and type of drinking water contamination that has been reported to result in violations of the SDWA.^10^ Using the USEPA Drinking Water Dashboard system, we obtained the total reported health-based violations for exceedances of maximum contaminant levels (MCLs), residual disinfectant levels, or treatment technique requirements for each major SDWA rule for all PWSs in the United States between 2010 and 2014. For completeness, we also report information on the total number of violations, which includes lack of monitoring and reporting by PWSs.

Having summarized the first pass removal efficiency for various contaminants indicated by the literature search and data analyses described above, we cross-tabulated the chemicals identified in the SDWA violations with the results of the literature review and data analyses described above to determine the number of incidents and size of the populations at risk for which POU treatment technologies with demonstrated efficacy could be used to mitigate exposures.

## Results and Discussion

### Literature review

Our search of the literature returned 3142 papers related to POU drinking water filtration. We excluded 3110 papers based on review of titles and abstracts and reviewed the full text of 32 papers. Of those papers, 18 were outside the scope of our analysis and were not considered further: 3 studies evaluated treatment technologies other than POU devices, 5 studies evaluated POU technologies not intended for residential application, 2 studies were conducted outside of the United States or Canada, 3 studies did not report filter performance data, and 5 studies addressed only microbiological contaminants.

The remaining 15 papers met the inclusion criteria and include 11 studies of POU filtration of inorganic contaminants and 4 of organic chemicals. Below, we summarize the filter types and corresponding removal efficiency for each of the contaminants reported in those papers along with relevant information on the settings and methods of the testing procedures. Detailed results from each of those publications are presented in Table S1 in Supplemental Material.

Of the 11 peer-reviewed studies of inorganic contaminants, one or more of the following elements were studied: Ag, Al, As, Co, Cr, Cu, Fe, Mn, Ni, Pb, Sb, and Zn. Four of the 11 inorganic studies evaluated POU performance for Pb either alone or in conjunction with other metals^11–14^ and 2 for Cu.^11,14^

Overall, faucet-mount or under-sink solid block activated carbon (SBAC) filters removed 80% to 99% of total Pb in 2 studies^12,14^ and 68% to 98% of total Cu in 1 study.^12^ The study by Deshommes et al included laboratory tests of 3 SBAC faucet-mount or under-sink filtration units as well as 1 pour-through pitcher. All filters removed more than 80% of dissolved Pb and up to 99% of particulate Pb. Notably, the pour-through filter reduced particulate Pb inconsistently with as little as 28% reduction. Similarly, the in-use study with more than 90 observations made in a penitentiary also showed the effectiveness of SBAC filters on both dissolved and particulate lead.^14^

In addition, one study evaluated SBAC performance for Mn reduction and found SBAC systems to be effective only up to approximately 50% of filter capacity, but filters containing cation exchange resin were effective in removing Mn at 60 to >99%.^15^ No corresponding NSF standard is applicable for Mn reductions, so consumers concerned about Mn in drinking water would not be able to identify a filter to address this metal.

Finally, 7 of the 11 POU filter studies measured reductions of As using POU filters.^13,16–21^ Reverse osmosis (RO) units were able to reduce on average 79 to >99% of As.^13,16–19,21^ Increasing As influent concentrations and water hardness decreased the effectiveness of RO units on As reductions.^16,19^

### POU filter study in Flint

**Figure 1.**
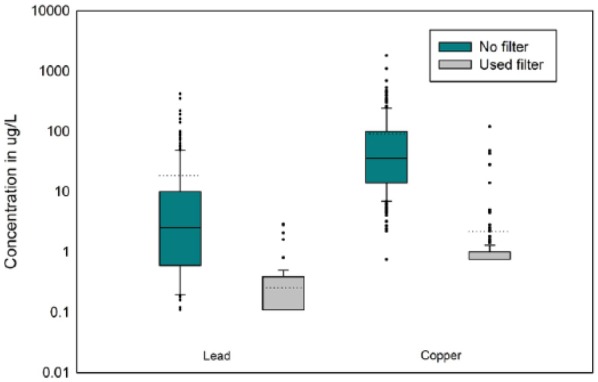
Boxplots of Pb and Cu concentrations from matched water samples collected in 208 homes and commercial locations in Flint, MI, January to March 2016. (The upper and lower parts of the boxplot are the 75th and 25th percentile values. The upper and lower whiskers are the 90th and 10th percentile values. The median is in the center of the box and the dotted line is the mean. Outlier points are shown as dots above or below the whiskers.)

The in-use effectiveness of NSF-approved faucet-mount filters for metals was tested in a study conducted by USEPA in residences and commercial locations in Flint, MI, during 2015.^8^ Table 1 presents summary statistics of the elemental concentrations in filtered and unfiltered water matched by location in the study (n = 208). The median Pb concentration in the samples collected without a filter was 2.5 µg/L, more than 20 times the median concentration of used filters (0.11 µg/L). Table 2 presents summary statistics on the differences between unfiltered and filtered water. The average difference in Pb concentration between filtered and unfiltered water (filtered – unfiltered) was –18.2 µg/L (SD = 49.0 µg/L) with a range of –418 to +0.7 µg/L (median = –2.3 µg/L). Elemental concentrations in the filtered water were significantly (*P* < .001) lower for all elements except Na and K, which were significantly higher (*P* < .001) in the filtered water than the unfiltered water. In addition, there was no statistically significant difference between used and new filters (n = 161) for any of the elements (*P* < .0001, rejecting the alternative hypothesis that the true difference between new and used filters was not equal to 0 for all elements). Figure 1 illustrates the highly skewed distributions of Pb and Cu measured in the used and no filter samples, but the much lower concentrations after filtration are apparent. Even in cases where the unfiltered water was as high as 419 µg/L of Pb, the highest filtered Pb concentration was 2.9 µg/L.

**Table 1.** Summary statistics of 208 pairs of water samples with and without a POU filter collected in Flint, MI, January to May 2016.

	Used filter						No filter					
	Mean (SD)	Min	P25	P50	P95	Max	Mean (SD)	Min	P25	P50	P95	Max
Al	0.051 (0.075)	0.009	0.009	0.009	0.200	0.330	0.129 (0.300)	0.009	0.021	0.043	0.370	2.62
Cd	0.452 (0.730)	0.061	0.061	0.061	2.00	2.00	0.641 (1.149)	0.061	0.061	0.140	2.00	12.0
Ca	22 (8)	0.2	22	26	28	32	27 (2.7)	0.24	26	27	29	32
Cr	1.25 (1.97)	0.20	0.20	0.20	5.00	5.00	1.35 (2.13)	0.20	0.20	0.25	5.00	14.1
Cu	2.2 (9.7)	0.75	0.75	0.75	2.3	120	92.0 (177)	0.75	14.3	36.0	349	1800
Fe	0.038 (0.069)	0.016	0.016	0.016	0.096	0.880	0.508 (2.86)	0.016	0.043	0.084	1.10	38.5
Pb	0.25 (0.35)	0.11	0.11	0.11	0.50	2.90	18.5 (49.0)	0.11	0.61	2.53	90.4	418
Mg	7.86 (2.00)	0.048	7.80	8.10	9.80	11.2	7.82 (0.688)	0.048	7.69	7.87	8.4	10.1
Mn	0.004 (0.006)	0.001	0.001	0.002	0.009	0.068	0.012 (0.048)	0.001	0.002	0.005	0.033	0.655
Ni	1.44 (2.25)	0.23	0.23	0.31	6.00	11.6	2.25 (2.99)	0.23	0.48	0.85	6.34	19.0
K	5.32 (10.4)	0.21	0.99	1.36	32.7	70	0.99 (0.09)	0.04	0.97	0.99	1.11	1.22
Na	9.31 (9.98)	0.98	4.80	5.39	30.3	80	5.16 (3.96)	4.1	4.6	4.8	5.27	52
Zn	0.051 (0.075)	0.009	0.009	0.009	0.200	0.330	0.129 (0.300)	0.009	0.021	0.043	0.370	2.62

Abbreviation: POU, point-of-use.

Units are in milligram per liter for all elements except Cr, Cu, Ni and Pb, which are in microgram per liter.

**Table 2.** Summary statistics of differences in elemental levels for 208 pairs of water samples with and without a POU filter collected in Flint, MI, January to May 2016.

Element	Mean (SD)	Min	Max	P25	Median	P95
Al	−0.1 (0.3)	−2.6	0.3	−0.044	−0.014	0.005
Cd	−0.2 (0.9)	−11.4	0.32	−0.1	0	0
Ca	−4.2 (8.2)	−29.3	21.4	−4.0	−1.0	2.0
Cr	−0.1 (0.6)	−9.1	0.48	−0.1	0	0
Cu	−89.8 (177)	−1799	47.25	−98.1	−34.3	−3.3
Fe	−0.5 (2.9)	−38.4	0.9	−0.184	−0.051	0
Pb	−18.2 (49.0)	−418	0.7	−9.9	−2.3	0
Mg	−0.04 (1.95)	−8.1	3.4	−0.1	0.3	1.9
Mn	−0.008 (0.048)	−0.647	0.064	−0.005	−0.001	0.004
Ni	−0.001 (0.002)	−0.019	0.006	−0.001	0	0
K	4.3 (10.4)	−0.7	69.0	0	0.4	31.7
Na	4.1 (9.9)	−32.4	75.2	0	0.6	25.0
Zn	−0.13 (0.51)	−6.5	0.09	−0.094	−0.024	0.009

Abbreviation: POU, point-of-use.

Units are in milligram per liter for all elements except Cr, Cu and Pb, which are in microgram per liter.

While NSF testing provides assessment of the potential effectiveness of POU filters, the data collected in Flint, MI, and released by USEPA provide a unique opportunity to assess in-use effectiveness of faucet-mount POU filters on Pb and other metals for hundreds of homes in that city. The data show substantial and statistically significant reductions in Pb and Cu with use of NSF-approved SBAC filters at the faucet.

The efficacy of SBAC POU filtration for Pb removal observed in Flint may have meaningful implications for managing risks of Pb in drinking water more broadly. A cross-sectional epidemiological study of 306 children found a strong association between Pb in home drinking water and elevated blood Pb levels (odds ratio [OR] = 4.66, confidence interval [CI] = 2.12-10.24).^22^ The 90th percentile concentrations of Pb in drinking water in study homes ranged from 4.51 to 10.06 µg/L, depending on the sample collection method employed. The 10th and 90th percentile blood lead levels were 0.77 and 1.31 µg/dL, respectively (geometric mean (GM) = 1.35 µg/dL, 95% CI = 1.27-1.43). In comparison, the 90th percentile concentration of Pb in filtered water sampled in Flint, MI, was 0.5 µg/L. The use of SBAC filters can reduce levels of Pb in drinking water and as a result may be able to reduce blood lead levels in those drinking water with elevated levels of Pb.

There are limitations to the Flint data, including the lack of repeated measures within homes over time, the lack of simultaneous pH measurements, and other indicators of hardness. However, the results support the hypothesis that SBAC filters can be effective for reducing levels of Pb and Cu (as well as many other elements) in drinking water with moderately elevated concentrations, as was the case in Flint. The Flint filter study also demonstrates that moderately scaled panel studies can provide a model for assessing additional contaminants of concern and POU technologies of interest. Although NSF testing provides an assessment of the potential effectiveness of POU filters, the data collected in Flint, MI, provided a unique opportunity to assess in-use effectiveness of faucet-mount POU filters on Pb and other metals for hundreds of homes in that city.

### Organic contaminants

While some SBAC faucet-mount POU filtration devices have NSF approval for trihalomethanes (THMs), no pour-through devices do. Three of the 4 studies of organic contaminants we reviewed examined the effectiveness of POU filters in reducing levels of disinfection by-products (DBPs), including THMs, in drinking water.^23–25^ Anumol et al^26^ examined removal efficiencies for 16 individual trace organic compounds (TORCs), including pharmaceuticals, pesticides, and other organic contaminants, by 2 refrigerator SBAC POU devices and a commercially available activated carbon pitcher. This study evaluated POU performance over the life of the filters and found that all were effective in reducing levels of TORCs initially by 70 to >90%. Refrigerator SBAC devices performed better than pitchers. Over time, filters performed better when challenged with groundwater than surface water, with high total organic carbon content with reduced flow occurring for several of the pitchers when surface water was the influent source.

Levesque et al^24^ conducted a field study of municipal tap water and found a POU filter pitcher device with activated carbon and ion exchange resin reduced THMs by approximately 90% and haloacetic acids (HAAs) by 50 to >70%. Another study evaluated additional POU pitchers and found them to remove >93% of trihalomethane-4 and 65 to >98% of HAA9.^25^

Scores of studies have shown adverse toxicological effects related to chlorination by-products,^4,24,27^ which are commonly found in drinking water. In general, there is a fairly large variation in the reduction efficiencies reported for contaminants treated by POU technologies tested in these studies. Only a small number of studies have evaluated effectiveness of POU filters on removing DBPs, such as THMs, and there are no pour-through filters with NSF certification demonstrating effectiveness of filters on DBPs.^28^ Some faucet-mount filters, particularly SBAC filters, are approved by NSF for reduction of THMs in tap water.

Turning now to other types of organic chemical contaminants, cases of drinking water contamination related to PFOA have been reported for numerous locations in the United States,^29–31^ and exposures via private wells have also been demonstrated,^32^ highlighting the risks of contaminants in both public and private water supplies. Only very recently was an NSF certification established for PFOA reduction with only 2 products approved as of August 2017.^28^ Prior to establishment of a PFOA/PFOS certification, USEPA and many state environmental and public health agencies have recommended use of POU filtration units for individuals living in areas known or suspected to have drinking water resources contaminated with PFOA or related compounds.^33,34^

Studies have also identified many classes of additional unregulated drinking water contaminants, including prescription medications, pesticides, and antimicrobials, that can make their way into drinking water systems.^35^ Health effects associated with relatively low-level exposures to mixtures of these compounds are not known, but health effects associated with contaminants of potential concern are under review by USEPA as part of its CCL program.^1^ In addition, the frequency of detection and magnitude of concentrations of certain unregulated contaminants are monitored under the UCMR. Despite the UCMR and drinking water regulations, concerns remain about the potential impacts of unregulated compounds in drinking water. Furthermore, many of these contaminants, including medications, pesticides, and flame retardants, can be treated with NSF-approved POU filters.

### US EPA SDWA violation data

Table 3 provides a summary of monitoring and reporting, as well as health-based SDWA violations, for inorganic and organic contaminant-related rules reported in the United States between 2010 and 2014. Violations related to monitoring and reporting are much more frequent than health-based violations as illustrated in the “total violations” columns. The number of health-based violations of the Lead and Copper Rule (LCR) was 290, potentially affecting 241 675 consumers. In comparison, when all violations, including lack of monitoring and reporting, are included, the total number of LCR violations increases to 6189, potentially affecting up to 14 461 735 consumers. This table also includes classes of POU treatment devices that have been approved by NSF to be effective in reducing contaminants associated with the corresponding SDWA violation. NSF-approved POU devices have the potential to mitigate risks from SDWA violations related to contaminants, such as lead, volatile organic compounds (VOCs), and DBPs, which have been shown to have hundreds, if not thousands, of violations across the United States in recent years.

**Table 3.** Data on health-based violations of inorganic and organic contaminant rules from USEPA ECHO database, 2010-2014.

SDW rule/category	Contaminant(s)	Health-based violations		Total violations		Type of POU to treat	NSF/ANSI certification(s)
		Number of violations	Size of population potentially affected	Number of violations	Size of population potentially affected		
LCR	Pb, Cu	290	241 675	6189	14 461 735	Carbon block	Standard 53
Chemical	SOC, VOCs, other inorganics,^a^ radionuclides	473	774 390	5101	17 704 590	Carbon block RO	Standard 53 Standard 58
DBPs	THMs, HAAs, chlorite, bromate	1442	8 837 437	8999	40 026 297	Carbon block	Standard 53
Arsenic	Arsenic	708	1 074 179	1685	3 550 325	RO Nanofiltration	Standard 58
Nitrates	Nitrate/Nitrite	669	454 532	9165	9 397 203	RO	Standard 58
						RO	Standard 58

Abbreviations: DBP, disinfection by-product; ECHO, Enforcement and Compliance History Online; HAAs, haloacetic acids; LCR, Lead and Copper Rule; RO, reverse osmosis; SOC, synthetic organic chemicals (includes 25 individual pesticides, ethylene dibromide, polychlorinated biphenyls, benzo(a)pyrene, di(ethylhexyl)-adipate, di(ethylhexyl)-phthalate, dioxin); THMs, trihalomethanes; USEPA, US Environmental Protection Agency; VOC = volatile organic compounds.

a Other inorganics include asbestos, Ba, Cd, Cr, F, Hg, Se, Sb, Be, cyanide, and Tl.

Some POU devices, such as carbon block filters, remove multiple categories of pollutants, including metals, VOCs, and particles. The efficacy of using POU filtration for drinking water contamination incidents has been demonstrated most recently for Pb in Flint, MI, drinking water. With the high incidence of monitoring and reporting violations and weak enforcement, POU filters with demonstrated effectiveness may be able to protect public health by treating water containing contaminants exceeding guidance values for the millions of individuals potentially affected by these violations.

Although there are limitations to these data, they provide an indication of the potential scope of drinking water contamination and the magnitude of potentially affected populations served by PWSs with violations that may not be reported to the public. In addition, the small number of health-based violations for some SDWA rules indicates potential gaps in assessment and/or enforcement programs.

Violations by definition occur after the discovery of a chemical contaminant in drinking water. As a result, elevated concentrations can also occur with no knowledge on the part of consumers or drinking water authorities. Perhaps even more concerning is the health risk for the more than 15 million US residents who obtain their drinking water from domestic private wells, which are not covered by federal regulations and have no centralized treatment or regular testing. These private wells are mostly located in rural areas, some of which are near septic and/or agricultural areas and can thus be vulnerable to chemical contamination due to nearby industrial sources or agricultural activities as well as PFOA. Perfluorooctanoic acid is an example where widespread contamination can go on unnoticed for extended periods of time. Point-of use filtration can be effective as a preventative measure in these situations. The utilization of POU filters can reduce exposures to these contaminants and should be considered as an added level of protection to increase the safety of drinking water from public or private sources.

## Conclusions

Despite promulgation and enforcement of SDWA regulations, concerns about drinking water quality are common in the United States, with 61% of Americans surveyed “worrying a great deal about polluted drinking water.”^36^ Although microbiological contaminants have historically been a focus of drinking water regulations, numerous studies have identified inorganic and organic chemical contaminants in drinking water, some of which enter drinking water supplies after the water has been tested at the drinking water treatment facility or are not removed by standard treatment methods. Finally, the extensive Pb contamination^2,37^ of drinking water in Flint, MI, in 2014 and in Washington, DC^6^ in 2005, as well as various cases of PFOA contamination across the United States, highlights the risks associated with poorly managed drinking water supplies in the United States and the potential role of POU faucet-mount filters.

Many types of residential water treatment devices available to consumers have undergone laboratory testing and often certification for efficacy against chemical contaminants in tap water. Laboratory evaluations have traditionally focused on the contaminants regulated under the SDWA in the United States. In recent years, these certifications include “emerging” contaminants of concern, including certain novel human carcinogens, industrial chemicals, pesticides, pharmaceuticals, personal care products, and flame retardants and DBPs. Some POU filters, such as SBAC, can remove multiple classes of contaminants, including both inorganic, such as Pb, and organic contaminants, such as flame retardants.

As previously mentioned, the SDWA requires only 2 contaminants be monitored at the tap, Pb and Cu, although others may be introduced into drinking water as it travels through the distribution system. A number of reports have highlighted that for many SDWA contaminants, there is weak enforcement of the required monitoring and reporting, as indicated by the large discrepancies between health-based violations and those that include monitoring and reporting violations. The timing of reported violations can often be much delayed from the actual health impacts in the community. There are a number of historical exposure scenarios in which a contaminant was introduced by the pipe network. This includes Pb in Flint, MI, Washington, DC, and elsewhere and tetrachloroethylene in parts of the northeastern United States. In all of these cases, the community was not aware of drinking water contamination for long periods of time, years in some cases.

While relatively few published studies have evaluated the effectiveness of POU filters in use, the studies we identified suggest that POU filters can be effective in reducing levels of chemical contaminants in drinking water. Future studies could evaluate the potential impacts of POU filtration on health endpoints, such as reducing blood lead levels or PFOA serum levels, in populations previously exposed to those contaminants in drinking water after consumers switch to filtered water.
